# Defects of full-length dystrophin trigger retinal neuron damage and synapse alterations by disrupting functional autophagy

**DOI:** 10.1007/s00018-020-03598-5

**Published:** 2020-08-04

**Authors:** Elisabetta Catalani, Silvia Bongiorni, Anna Rita Taddei, Marta Mezzetti, Federica Silvestri, Marco Coazzoli, Silvia Zecchini, Matteo Giovarelli, Cristiana Perrotta, Clara De Palma, Emilio Clementi, Marcello Ceci, Giorgio Prantera, Davide Cervia

**Affiliations:** 1 Department for Innovation in Biological, Agro-Food and Forest Systems (DIBAF), Università degli Studi della Tuscia, largo dell’Università snc, 01100 Viterbo, Italy; 2 Department of Ecological and Biological Sciences (DEB), Università degli Studi della Tuscia, largo dell’Università snc, 01100 Viterbo, Italy; 3 Section of Electron Microscopy, Great Equipment Center, Università degli Studi della Tuscia, largo dell’Università snc, 01100 Viterbo, Italy; 4 Department of Biomedical and Clinical Sciences “Luigi Sacco” (DIBIC), Università degli Studi di Milano, via G.B. Grassi 74, 20157 Milano, Italy; 5 Department of Medical Biotechnology and Translational Medicine (BioMeTra), Università degli Studi di Milano, via Luigi Vanvitelli 32, 20129 Milano, Italy; 6 Unit of Clinical Pharmacology, University Hospital “Luigi Sacco”-ASST Fatebenefratelli Sacco, via G.B. Grassi 74, 20157 Milano, Italy; 7 Scientific Institute IRCCS “Eugenio Medea”, via Don Luigi Monza 20, 23842 Bosisio Parini (LC), Italy

**Keywords:** Apoptosis, Autophagy, Dystrophin, Neurodegeneration, Retina neurons, Visual function

## Abstract

Dystrophin (dys) mutations predispose Duchenne muscular disease (DMD) patients to brain and retinal complications. Although different dys variants, including long dys products, are expressed in the retina, their function is largely unknown. We investigated the putative role of full-length dystrophin in the homeostasis of neuro-retina and its impact on synapsis stabilization and cell fate. Retinas of *mdx* mice, the most used DMD model which does not express the 427-KDa dys protein (Dp427), showed overlapped cell death and impaired autophagy. Apoptotic neurons in the outer plexiform/inner nuclear layer and the ganglion cell layer had an impaired autophagy with accumulated autophagosomes. The autophagy dysfunction localized at photoreceptor axonal terminals and bipolar, amacrine, and ganglion cells. The absence of Dp427 does not cause a severe phenotype but alters the neuronal architecture, compromising mainly the pre-synaptic photoreceptor terminals and their post-synaptic sites. The analysis of two dystrophic mutants of the fruit fly *Drosophila melanogaster*, the homozygous *Dys*^E17^ and *Dys*^EP3397^, lacking functional large-isoforms of dystrophin-like protein, revealed rhabdomere degeneration. Structural damages were evident in the internal network of retina/lamina where photoreceptors make the first synapse. Both accumulated autophagosomes and apoptotic features were detected and the visual system was functionally impaired. The reactivation of the autophagosome turnover by rapamycin prevented neuronal cell death and structural changes of mutant flies and, of interest, sustained autophagy ameliorated their response to light. Overall, these findings indicate that functional full-length dystrophin is required for synapsis stabilization and neuronal survival of the retina, allowing also proper autophagy as a prerequisite for physiological cell fate and visual properties.

**Electronic supplementary material:**

The online version of this article (10.1007/s00018-020-03598-5) contains supplementary material, which is available to authorized users.

## Introduction

Dystrophin (dys) is the largest human gene that generates, through multiple, tissue-specific promoters, a family of distinct isoforms of cytoskeleton-associated proteins named by their molecular weight [1]. The longest dys product, a 427-KDa protein (Dp427), is a crucial component of the dystrophin-glycoprotein complex (DGC) that bridges the inner cytoskeleton and the extracellular matrix of myofibers stabilizing the sarcolemma. Mutations of dys lead to Duchenne muscular dystrophy (DMD) a recessive X-linked, severe, progressive muscle degenerative disease that preferentially affects male children [2]. Improvements in patient care and disease management slowed DMD progression, but current treatments cannot stop the relentless loss of muscle tissue and function, which leads to premature death [2]. Dystrophin and its variants are also present in extra muscular tissues, e.g., in the nervous systems [3, 4]. In this respect, several pieces of evidence point to brain dysfunction as an intrinsic feature of DMD, disputing our view of the disease as an exclusively neuromuscular one [5, 6]. Accordingly, clinical observations showed that DMD is often accompanied by neurocognitive symptoms, epilepsy, and learning disabilities, suggesting that the absence of dystrophin affects neuronal excitability and development [5–10].

Since dys gene is evolutionarily well conserved, a number of animal models of DMD have been described including mouse, dog, zebrafish, the nematode *Caenorhabditis elegans*, and the fruit fly *Drosophila melanogaster* [11–13]. In the last decade, several studies in vertebrate and invertebrate model systems highlighted the role of DGC and/or dystrophin variants at both peripheral and central synapses; as for instance, in the development of nervous structures, including the retina, in the maturation of neurotransmitter-receptor complexes and in the regulation of neurotransmitter release, in the modulation of excitatory/inhibitory signaling of neurons and in neuronal density of the brain [7, 8, 14–30]. In the human nervous system, large changes in the expression of multiple dystrophin isoforms occur throughout life cycle especially between the fetal and adult brain [3], thus suggesting the cell-type specific function of distinct dystrophin variants across nervous regions.

Several neurodegenerative conditions have manifestations in the eye, and ocular symptoms often precede conventional diagnosis [31]. Accordingly, in DMD patients, post-receptor mechanisms underlying scotopic and photopic vision and ON- and OFF-pathway responses measured by electroretinography (ERG) may be altered (also depending on the site of dys mutation), suggesting that dystrophins are required for normal electrophysiology and for proper function of luminance and red-green cone opponent mechanisms in the human retina [9, 32–37]. Although previous studies on mammalian retinas proposed that dystrophins act predominantly at photoreceptor terminals, it was demonstrated later that different dys products, i.e., Dp427, Dp260, Dp140, and Dp71 are expressed in the outer nuclear layer (ONL), the inner nuclear layer (INL), and the ganglion cell layer (GCL) [32, 34, 35, 38–46]. Despite this observation their functional role is largely unknown.

Under physiological and pathological conditions, there are many common ways for neurons to die or survive, although their complexity make them sensitive/susceptible to unique events [47]. Apoptosis participates in the death of retinal neurons, but the catabolic pathway autophagy has been recognized also to be involved with both synergistic and antagonistic roles [48–50]. Recently, we have demonstrated a cross-talk between apoptosis and autophagy in distinct retinal cell populations of mouse which was altered by pathological conditions favoring apoptosis, but it may be re-equilibrated by autophagy-stimulating factors, thus restoring retinal functions [51, 52]. Although autophagy is required for the maintenance of skeletal muscle homeostasis and there is a causal relationship between DMD pathogenesis and dysfunctional autophagy [53–56], no evidence exists on the involvement of dystrophins in apoptosis/autophagy balance of neuronal cells.

In this study, we sought to investigate the putative role of full-length dystrophin in the homeostasis of neuro-retina and its molecular impact on synapsis stabilization and cell fate. We demonstrated for the first time that neuronal populations of the retina displayed apoptotic features and impaired autophagy in dystrophic mouse and *D. melanogaster* models expressing defective full-length dystrophins, with the first synapse photoreceptor/bipolar cells being highly affected. Moreover, we showed that boosting autophagy preserved visual function counteracting apoptotic-induced neurodegeneration and synapsis destabilization. These results provide proof of concept of the therapeutic potential of autophagy tuning for defective dystrophin-induced neurodegeneration.

## Materials and methods

### Reagents

The primary antibodies, including their suppliers, are listed in Table 1. Bovine serum albumin (BSA), normal goat serum, Alexa Fluor secondary antibodies, and fluorescent phalloidin were purchased from Life Technologies (Monza, Italy). Fluoroshield Mounting Medium containing 4′,6-diamidine-2′-phenylindole dihydrochloride (DAPI) was obtained from Abcam (Cambridge, UK). Cacodylate buffer, osmium tetroxide, Agar 100 resin/propylene oxide, and Agar 100 resin were obtained from Electron Microscopy Sciences (Hatfield, PA, USA). All other chemicals including rapamycin (50 mM in ethanol) were from Sigma-Aldrich (St. Louis, MO, USA).

**Table 1:** Primary antibody information

Antibody	Host	Diluition*	Source	Cat. No
AffiniPure Fab Fragment IgG (H + L)	Goat	1:50	Jackson ImmunoResearch	111-007-003
Calbindin-D-28 K	Mouse	1:3000	Sigma–Aldrich	C9848
Cleaved caspase 3	Rabbit	1:400 (M)/ 1:500 (D)	Sigma–Aldrich/ Cell Signaling Technology	C8487/ 9664
CtBP2	Mouse	1:2000	BD Biosciences	612044
Dab-1	Rabbit	1:300	Sigma–Aldrich	SAB4503448
Fluorescein AffiniPure Fab Fragment IgG (H + L)	Goat	1:400	Jackson ImmunoResearch	111-097-003
GAT-1	Rabbit	1:400	Merck Millipore	AB1570
GFAP	Mouse	1:400	Sigma–Aldrich	G3893
LC3	Rabbit	1:100 (M)/ 1:100 (D)	Sigma–Aldrich	L8918/ ab128025
MAb115A10	Mouse	1:200	Shinobu C. Fujita (Japan)	
mGluR6	Rabbit	1:100	Novus Biologicals	NB300-189
p62/SQSTM1	Rabbit	1:200	Sigma–Aldrich	P0067
PKC	Mouse	1:200	Sigma–Aldrich	P5704
β-Tubulin III	Mouse	1:400	Sigma–Aldrich	T8660

*CtBP2* C-terminal-binding protein 2, *Dab-1* disabled-1, *GAT-1* γ-aminobutyric acid transporter-1, *GFAP* glial fibrillary acidic protein, *LC3* light-chain 3, *mGluR6* metabotropic glutamate receptor 6, *PKC* protein kinase C, *SQSTM1* Sequestosome-1

*D Drosophila melanogaster, *M* mouse

### Mice

Dp427 deficient male mice (C57BL/10ScSn-Dmd^mdx^, *mdx*) and their wild-type (wt) littermate controls with the background of *mdx* (C57BL/10ScSnJ) were obtained (4 weeks of age) from Jackson Laboratories (Bar Harbor, ME, USA). Animals were housed in a regulated environment (23 ± 1 °C, 50 ± 5% humidity) with a 12-h light/dark cycle (lights on at 08.00 a.m.), and provided with food and water *ad libitum*. Young adult mice (12–20 weeks) were euthanized at the indicated ages and their eyes enucleated. Generally, the one eye was processed for histology and electron microscopy while the other eye for immunostaining analyses.

### *Drosophila melanogaster* stocks and genetics

Dystrophic mutants used in this study were *Dys*^E17^ [57, 58] and P{EP}*Dys*^EP3397^ [59]. Oregon-R was used as wt strain. All stocks were obtained from Bloomington Drosophila Stock Center (Indiana University Bloomington, IN, USA): #63047 (*Dys*^E17^), #17121 *(Dys*^EP3397^), and #5 (Oregon-R). Allele description of *Dys*^E17^: point mutation induced by ethyl methanesulfonate on chromosome 3, 92A10, 3R:19,590,458. 19,590,458, causing a nucleotide change C19590458T and consequently the amino acid change Q2807term|Dys-PA. Third chromosome alleles were balanced with the TM6,Tb balancer chromosome. Allele description of *Dys*^EP3397^: insertion derived by TE mobilization using the transgenic transposon P{EP} on chromosome 3, 92A5, 3R:19,464,148. 19,464,148. Third chromosome alleles were balanced with the TM6,Tb balancer chromosome.

### Dietary condition, mating procedure, and rapamycin feeding of *Drosophila melanogaster*

Using published protocols [60], flies were raised on a standard corn meal agar food (pH 5.5) at 25 °C. We prepared fly food as follows: for 1.2 l of water 100 g of live yeast, 110 g of glucose, 100 g of corn meal and 8 g of agar were added and dissolved in warm water. The mixture was autoclaved and allowed to cool down slowly. The fungicide Nipagin (3 g dissolved in 16 ml of absolute ethanol) was added when the temperature reached approximately 50 °C, and the mixture was then dispensed into vials. Populations of adult flies (3 days old) were placed in vials (15 females and 10 males) for mating and eggs laying. After 3 days, mating flies were removed and, at around day 10 from mating, adults emerged from their pupal cases (eclosion). The developed animals from the pupae, i.e., young adults (1–2 days of adult age), were sampled for analysis. When indicated, flies were reared on either the standard medium or diet supplemented with rapamycin. Standard diet with rapamycin vehicle was used as control (mock treatment). Behavioral deficits (reduced body-wall contractions, mouth-hook movements and responses to mechanical stimuli) have been recently reported in rapamycin fed *D. melanogaster* larvae (Canton S strain) [61]. Additionally, a dose–response relationship between rapamycin feeding and time to pupation was observed, with 10–100 μM dosages taking longer to pupate. This impact on larval development is likely due to synaptic alterations within the central nervous system. Thus, in a preliminary experiment we tested increasing concentrations of rapamycin in our fly system. In the presence of 500, 200, and 100 μM rapamycin eggs (Oregon-R strain) did not hatch after 3 days from deposition, while an evident decrease of eclosion was achieved at 10, 1, and 0.1 μM (data not shown). In contrast, we did not find any significant change of egg-to-adult development at 10 nM rapamycin when compared to mock control, suggesting no evident effects on larval viability. Subsequent experiments were carried out using rapamycin at a final concentration of 10 nM.

### Histochemistry and immunofluorescence confocal microscopy

Using published protocols [62–64], mice eye-cups were immersion-fixed for 2 h in 4% paraformaldehyde in 0.1 M phosphate buffer (PB) at 4 °C, transferred to 25% sucrose in PB, and stored at 4 °C for at least 12 h. Drosophila heads were immersion-fixed overnight in 4% paraformaldehyde in PB at 4 °C, transferred to 12% sucrose in PB, and stored at 4 °C for at least 24 h [60]. Sections (10 µm thick) were obtained by a cryostat, mounted onto positive charged slides and stored at − 20 °C until use. For histochemistry, toluidine blue (to discriminate the different retinal layers) and fluorescent phalloidin (to detect rhabdomere morphology) was used in eye sections of mice and *D. melanogaster*, respectively. Sections were acquired and analyzed by bright field imaging using a Zeiss microscope (Axioskop 2 plus, Carl Zeiss, Oberkochen, Germany) equipped with the Axiocam MRC photocamera and the Axiovision software. For immunostaining detection in mice and *D. melanogaster*, sections were washed in PB and then pre-incubated for 30 min at room temperature with 5% BSA and 10% of normal goat serum in PB containing 0.5% Triton X-100. Pre-treated sections were incubated overnight at 4 °C with the primary antibodies listed in Table 1 diluted in PB containing 0.5% Triton X-100. When indicated, mice sections were also processed for double-label staining. Double-labeling experiments with anti-cleaved caspase 3, anti-light-chain 3 (LC3), anti-γ-aminobutyric acid transporter-1 (GAT-1), anti-disabled-1 (Dab-1), and anti-metabotropic glutamate receptor 6 (mGluR6) antibodies, which are all made in rabbit, were performed as previously published [51, 52, 65]. Briefly, sections were first incubated with anti-LC3 antibody for 3 h at room temperature and then in anti-rabbit fluorescein conjugated Fab fragment antibody (Table 1) for 1.5 h at room temperature. Subsequently, the slides were incubated in anti-rabbit unlabeled Fab fragment antibody (Table 1) overnight at 4 °C and then with the other primary antibodies for 3 h at room temperature. Following washes in PB, the sections were incubated in the appropriate Alexa Fluor secondary antibodies in PB for 1.5 h at room temperature. The slides were coverslipped with Fluoroshield Mounting Medium containing DAPI for nuclei detection. Incubation in secondary antibody alone was performed as negative control. Images were acquired by a Zeiss LSM 710 confocal microscope (Carl Zeiss, Oberkochen, Germany). Mouse retina thickness was evaluated on at least 10 Sects. (3 representative images) for each retinas, homogenously distributed following a nasotemporal sequence. The number of ganglion cells was calculated using the linear cell density (cells per 300 μm). For the analysis of cleaved caspase 3, LC3, and p62 immunostaining in retinas of mice, 3 representative images were selected for each retina section (at least 3 different sections for each retina). The procedure on *D. melanogaster *was carried out on the single images of each eye section (at least 2 different sections for each eye). Each image was converted to grayscale and normalized to background using Adobe Photoshop (Adobe Systems, Mountain View, CA, USA). Mean gray levels were then measured in the selected areas [52, 66].

### Transmission electron microscopy (TEM)

Using published protocols [67, 68], mice eye-cups and *D. melanogaster* heads samples were fixed overnight at 4 °C with a 2.5% (v/v) glutaraldehyde and 2% (v/v) paraformaldehyde in 0.1 M cacodylate buffer, pH 7.2. After 3 washes in the same buffer at 4 °C, for a total of 1 h, samples were post-fixed with 1% (v/v) osmium tetroxide in 0,1 M cacodylate buffer, pH 7.2 for 2 h at 4 °C. Specimens were washed 3 times in the same buffer for a total of 45 min, at 4 °C, and then dehydrated in a graded ethanol series, followed by two steps for 10 min each, at 4 °C in propylene oxide. Samples were then infiltrated with mixtures of Agar 100 resin/propylene oxide in different percentages. At the end of the procedure, samples were embedded in pure Agar 100 resin and let to polymerize for 2 days at 60 °C. Resin blocks were cut with Reichert Ultracut ultramicrotome using a diamond knife. Ultrathin Sects. (60–80 nm) (Leica Microsystems, Wetzlar, Germany) were collected on copper grids, stained with uranyl acetate and lead citrate, and observed with a JEOL 1200 EXII electron microscope (Jeol, Tokyo, Japan). Micrographs were captured by the Olympus SIS VELETA CCD camera equipped with iTEM software (Olympus, Tokyo, Japan). Quantitative analysis of autophagosome-like compartments was performed on selected TEM images of mice retinas. At least 7 representative images at same magnification were examined from each retina section (at least 3 different sections for each retina).

### Scanning electron microscopy (SEM)

*D. melanogaster* heads were fixed and dehydrated as described for TEM. Samples were dried by the critical point method using CO_2_ in a Balzers Union CPD 020 apparatus (Balzers, Liechtenstein). Then samples were attached to aluminum stubs using a carbon tape and sputter-coated with gold in a Balzers Union MED 010 unit. The observations were made by a JSM 6010LA electron microscope (Jeol, Tokyo, Japan).

### Climbing assay of *Drosophila melanogaster*

Geotaxis was assessed using a climbing assay as previously published with minor modifications [69]. Young adult flies were collected shortly after eclosion (1–2 days) and separated into cohorts (empty vials) consisting of 10 flies for each genotype. A horizontal line was drawn 18 cm above the bottom of the vial. After a 10-min rest period, the flies were tapped to the bottom of the vials, and the number of flies that climbed up to the 18-cm mark after 120 s was recorded as the percentage success rate. A camera was recording fly movement during the experiment. Each trial was performed three times, at 1-min intervals, and the results were averaged.

### Response to light of *Drosophila melanogaster*

Phototaxis assay of young adults flies (1–2 days after eclosion) was conducted as reported before with minor modifications [70]. Briefly, a plastic vial (2.5 × 9.5 cm) with flies was inserted and connected to a glass tube (2.5 × 20 cm) by transparent tape. The transparent apparatus (2.5 × 28 cm) was placed horizontal and perpendicular to the light source. The directional light source from one side, placed horizontal 15 cm away from the tube, acted as an attractant for the flies. In a dark room 10–20 flies were independently introduced in the apparatus and left separately for 30 min. This allowed adaptation of the flies to darkness. The apparatus was then gently pounded down to place the flies at opposite end from the light. The light was then turned on and a timer was started. A camera was recording fly behavior and their movement toward the light source during the experiment (1 min). For the analysis, the flies were counted every 10 s for each half of the apparatus, i.e., 0–10 cm (the chamber nearest to origin) and 10–28 cm (the chamber furthest to origin). The measurements of navigation strategies of *D. melanogaster* toward the light source were adapted from previous reports [71].

### Statistics

Statistical significance of raw data between the groups in each experiment was evaluated using unpaired Student’s t test (single comparisons) or one-way ANOVA followed by the Tukey post-test (multiple comparisons). The navigation behavior of flies was analyzed by two-way ANOVA and Sidak’s multiple comparisons test. A *p* value ≤ 0.05 is considered statistically significant. Data belonging from different experiments were represented and averaged in the same graph. The GraphPad Prism software package (GraphPad Software, San Diego, CA, USA) was used. The results were expressed as means ± SEM of the indicated n values.

## Results

### Apoptosis and autophagy in retinas of *mdx* mice

**Fig. 1:**
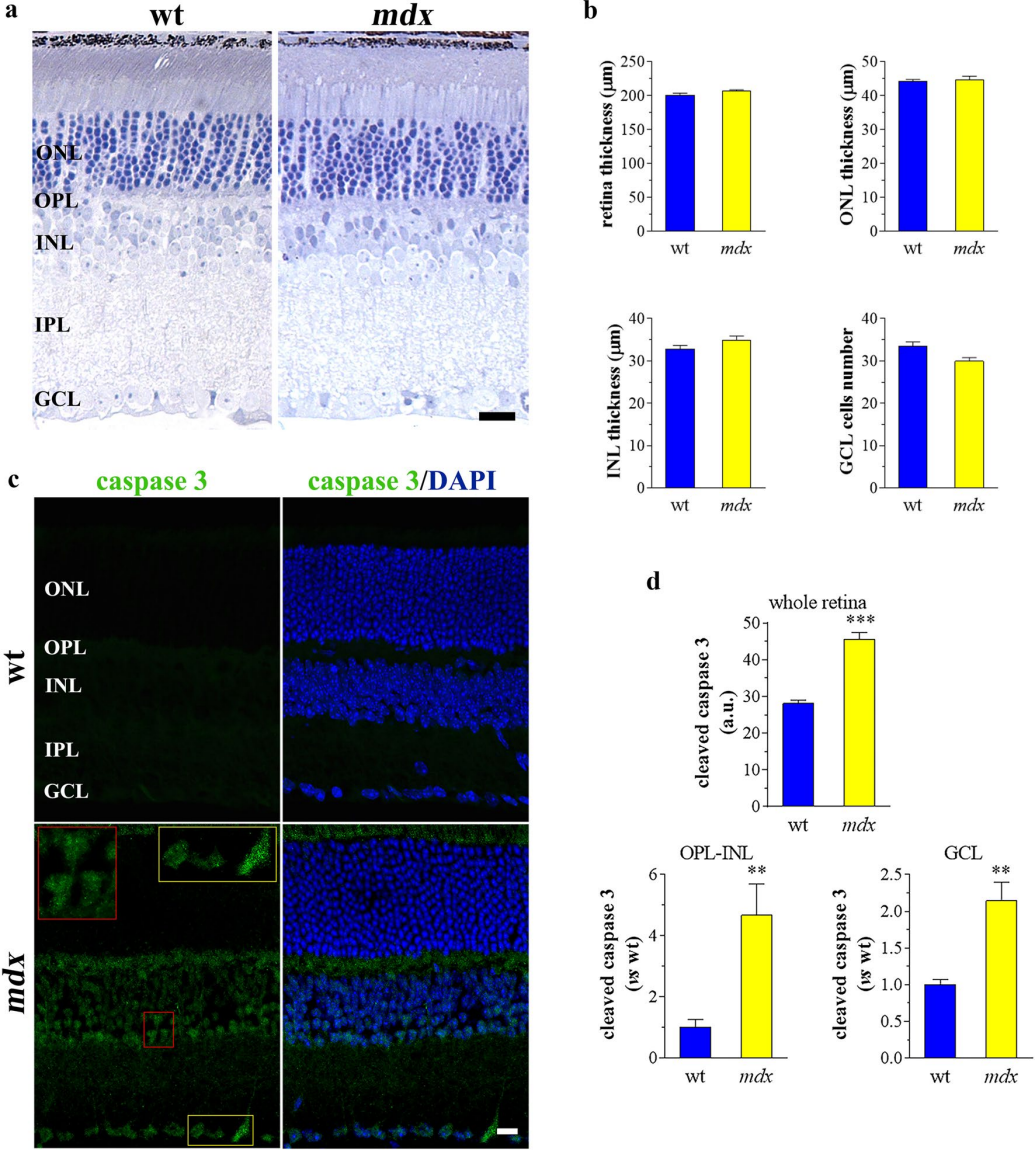
Apoptosis in dystrophic mouse retinas. **a** Morphological analysis by toluidine blue staining of sections from wt and *mdx* retinas. Scale bar: 20 µm. **b** The thickness of the whole retina (between the inner limiting membrane and pigment epithelium), ONL, INL, and the number of cells in GCL (cells per 300 μm of retinal lenght) were measured. **c** Confocal immunofluorescence imaging of cleaved (active) caspase 3 in wt and *mdx* retinas. DAPI was used for nuclei detection. Scale bar: 20 μm. Inserts represent enlarged image details. **d** Quantitative analysis of caspase 3 immunofluorescence in the whole retina, OPL-INL, and GCL. Results are expressed as arbitrary units (a.u.) or as fold change of wt. ***p < 0.0001 and **p < 0.001 vs wt (unpaired Student’s t test). Images and quantitative data are representative of *n* = 6 retinas from different mice

The “standard” *mdx* mouse, the most widely used model of DMD, does not express Dp427 since it has a nonsense point mutation (C-to-T transition) in exon 23 of dys, which creates a premature stop codon [12]. While lacking Dp427 in the retina, these animals express all other dys products [44], such that this model is useful for analysis of Dp427 actions in the eye [39, 44]. To observe the retinal morphology of young adult male mice (12–20 weeks) in the absence of Dp427 we used toluidine blue staining. Retina of *mdx* animals and their littermate wt controls exhibited normal arrangement of retinal cell layers (Fig. 1a). In addition, retina thickness of the whole retina, defined as the distance from the retinal pigment epithelium layer to the GCL, did not change in *mdx* mice compared to that of the wt mice. Similar results were obtained in ONL and INL (Fig. 1b). Accordingly, in *mdx* retinas no evident modifications of ganglion cell number were achieved. To evaluate neuronal cell death, the immunofluorescence of cleaved (active) caspase 3, an efficient marker of retinal apoptosis [51, 52], was evaluated by confocal microscopy. As shown in Fig. 1c,d, active caspase 3 was expressed in retinal sections of *mdx* mice, with a specific localization in cells of the outer plexiform layer (OPL), INL, and GCL, while rare or no signal was observed in the ONL and the inner plexiform layer (IPL). In contrast, active caspase 3 staining was almost undetectable in retinal sections from wt mice.

**Fig. 2:**
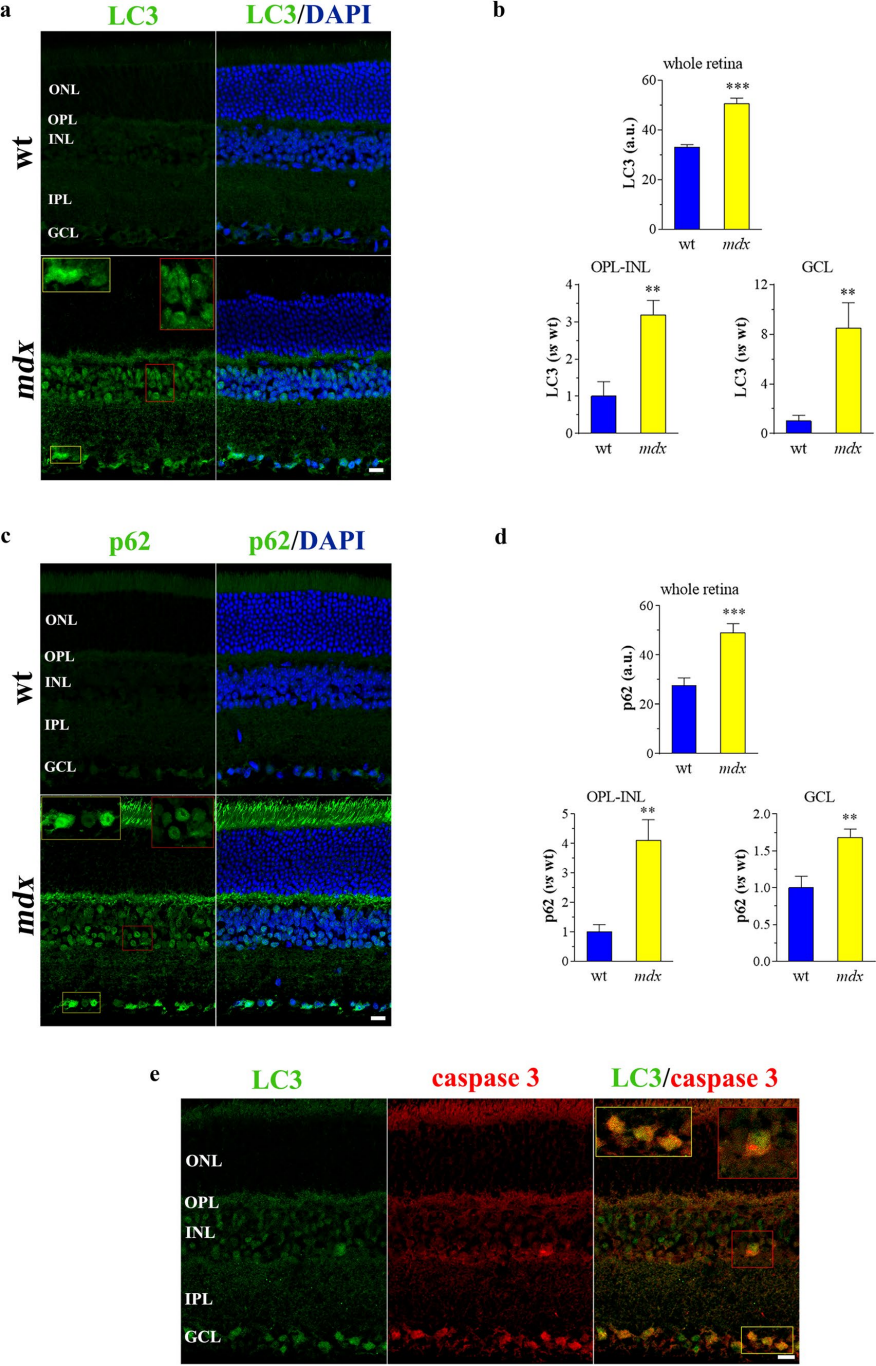
Autophagy in dystrophic mouse retinas. Confocal immunofluorescence imaging of LC3 **(a)** and p62 **(c)** in wt and *mdx* retinas. DAPI was used for nuclei detection. Scale bar: 20 μm. Insets represent enlarged image details. Quantitative analysis of LC3 **(b)** and p62 **(d)** immunofluorescence in the whole retina, OPL-INL, and GCL. Results are expressed as arbitrary units (a.u.) or as fold change of wt. ***p < 0.0001 and **p < 0.001 vs wt (unpaired Student’s t test). **e** Double-label immunofluorescence in *mdx* retinas using antibodies directed to LC3 and cleaved (active) caspase 3. Scale bar: 20 μm. Insets represent enlarged image details. Images and quantitative data are representative of *n* = 6 retinas from different mice

Changes in autophagic function can be detected by immunofluorescence analysis of the autophagic protein LC3 [72]. As shown in Fig. 2a, faint profiles of LC3 staining were observed in retinas of wt mice. In contrast, *mdx* retinas displayed an intense LC3 signal. The immunofluorescence was localized mainly to the OPL-INL and to the GCL, with scattered puncta also visible in the IPL. LC3 immunostaining in the INL and GCL was characterized by strongly fluorescent aggregates in the cell bodies, which were particularly densely packed within the immunostained profiles in the GCL. A quantitative analysis of LC3 immunofluorescence intensity confirmed a significant increase of LC3 expression in the absence of Dp427 (Fig. 2b). The punctate appearance of LC3 labeling revealed its presence in accumulating autophagosomes awaiting lysosomal degradation. The measurement of p62, a cargo receptor for ubiquitinated substrates degraded during autophagy, is a useful method to distinguish whether autophagosome accumulation is due to autophagy induction or rather to the inhibition of autophagy steps [72]. In control retinas, p62 displayed a widespread staining which was barely visible (Fig. 2c). Of note, p62 immunofluorescence markedly increased in *mdx* mice indicating an impairment of autophagy (Fig. 2c, d). Similar to LC3 pattern, p62 localized to the OPL, INL, and GCL where staining profiles were characterized by intense immunolabeled aggregates. To visualize better the relationship between apoptosis and autophagy, double-label immunofluorescence of active caspase 3 and LC3 was performed in retinal sections from *mdx* mice. As shown in Fig. 2e, caspase 3 immunostaining fully overlapped with the bright puncta pattern of LC3 immunoreactivity, thus indicating the close association of cell damage and impaired autophagy in retinal neurons lacking Dp427.

Gliosis is characterized as a hallmark of retinal degeneration, and this may occur through to release of pro-inflammatory mediators [73]. As shown in the Suppl. Figure 1, glial fibrillary acidic protein (GFAP)-immunoreactivity, an established indicator of reactive glia cells, was localized typically to the inner retina of normal samples and represents the cell body and processes of retinal astrocytes. Similar GFAP expression profiles were achieved in *mdx* and control retinas, supporting the notion that *mdx* retinas are not severely impaired.

### Retinal neurons with impaired autophagy in *mdx* mice

**Fig. 3:**
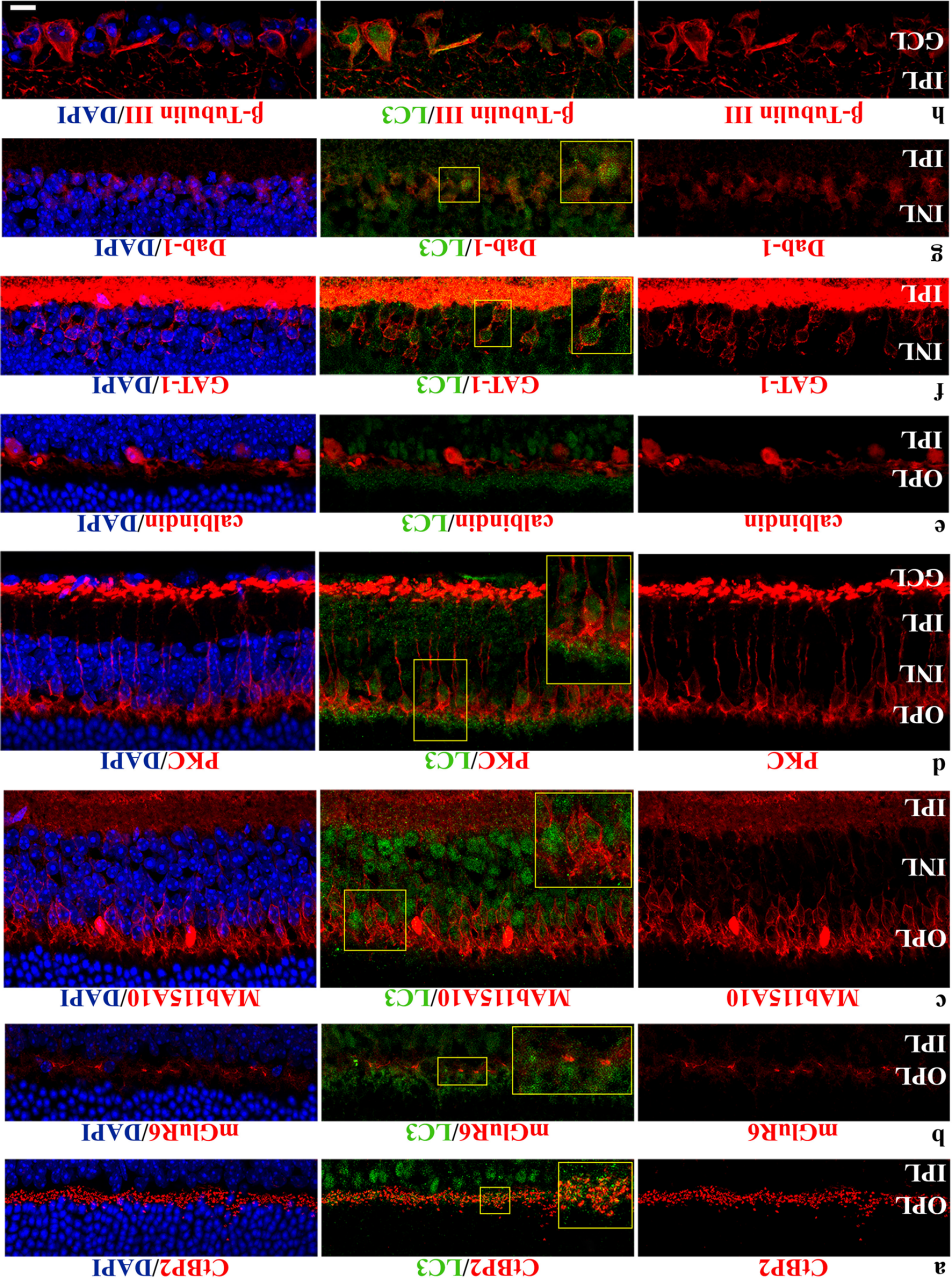
Autophagy in neuronal populations of dystrophic mouse retinas. Confocal double-label immunofluorescence imaging of LC3 and CtBP2 **(a)**, mGluR6 **(b)**, MAb115A10 **(c)**, PKC **(d)**, calbindin **(e)**, GAT-1 **(f)**, Dab-1 **(g)**, and ß-tubulin III **(h)** in *mdx* retinas. DAPI was used for nuclei detection. Scale bar: 20 μm. Inserts represent enlarged image details. Images are representative of *n* = 6 retinas from different mice

To gather some information about the cellular types expressing autophagy markers in damaged retinas of young adult *mdx* mice, the antibody directed to LC3 was used in double-label immunofluorescence experiments. Synaptic ribbons are organelles found at presynaptic active zones of sensory neurons that generate sustained graded electrical signals in response to stimuli, including retinal photoreceptor cells [74]. In particular, photoreceptor axonal terminals form ribbon synapses in the OPL, which connect photoreceptor pre-synapses with the dendritic terminal of both rod/cone bipolar cells and horizontal cells. As shown in Fig. 3a, the synaptic ribbon marker C-terminal-binding protein 2 (CtBP2) overlapped or was observed adjacent to LC3 puncta. In vertebrate retina, it is reasonable to assume that some bipolar cells are post-synaptic only to rods, others only to cones, and still others receive mixed rod-cone input [74, 75]. Of interest, while cone bipolar cells are either ON or OFF, rod bipolar cells are all of the ON variety [74, 75]. In *mdx* retinas, bipolar dendritic tips of OPL were stained with an antibody anti-mGluR6, which is specifically expressed at the post-synaptic site of bipolar cells and is responsible for ON responses in both the rod and cone systems. We found that mGluR6 signal localized adjacent to LC3 clusters (Fig. 3b). In addition, the intensely immunolabeled MAb115A10 (an antigen expressed by ON-type bipolar cells, which include ON-cone bipolar cells and rod bipolar cells) and protein kinase C (PKC) (a marker of rod bipolar cells) positive cells in INL were almost superimposable within LC3 immunoreactive profiles (Fig. 3c–d), although we found that some LC3 positive cells in mid-distal INL where not MAb115A10 positive. In contrast, no LC3 clusters accumulated at INL horizontal cells, immunostained with calbindin (Fig. 3e). Regarding amacrine cells expressing LC3 immunoreactivity, double immunolabeling was performed using an antibody directed to GAT-1, which identifies the majority of GABAergic amacrine cells. Different LC3-positive cells in the proximal INL of *mdx* retinas displayed also GAT-1 immunoreactivity on their membrane (Fig. 3f). Similarly, Dab-1 positive cells, indicating glycinergic AII amacrine cells, are also positive for LC3 (Fig. 3g). Finally, to evaluate the presence of LC3 puncta in GCL, we used an antibody directed to ß-tubulin III, which is expressed by ganglion cells. As shown in Fig. 3h, LC3 immunostained profiles of *mdx* retinas were also ß-tubulin III immunolabeled, and vice versa.

### Ultrastructural analysis *mdx* mice retinas

**Fig. 4:**
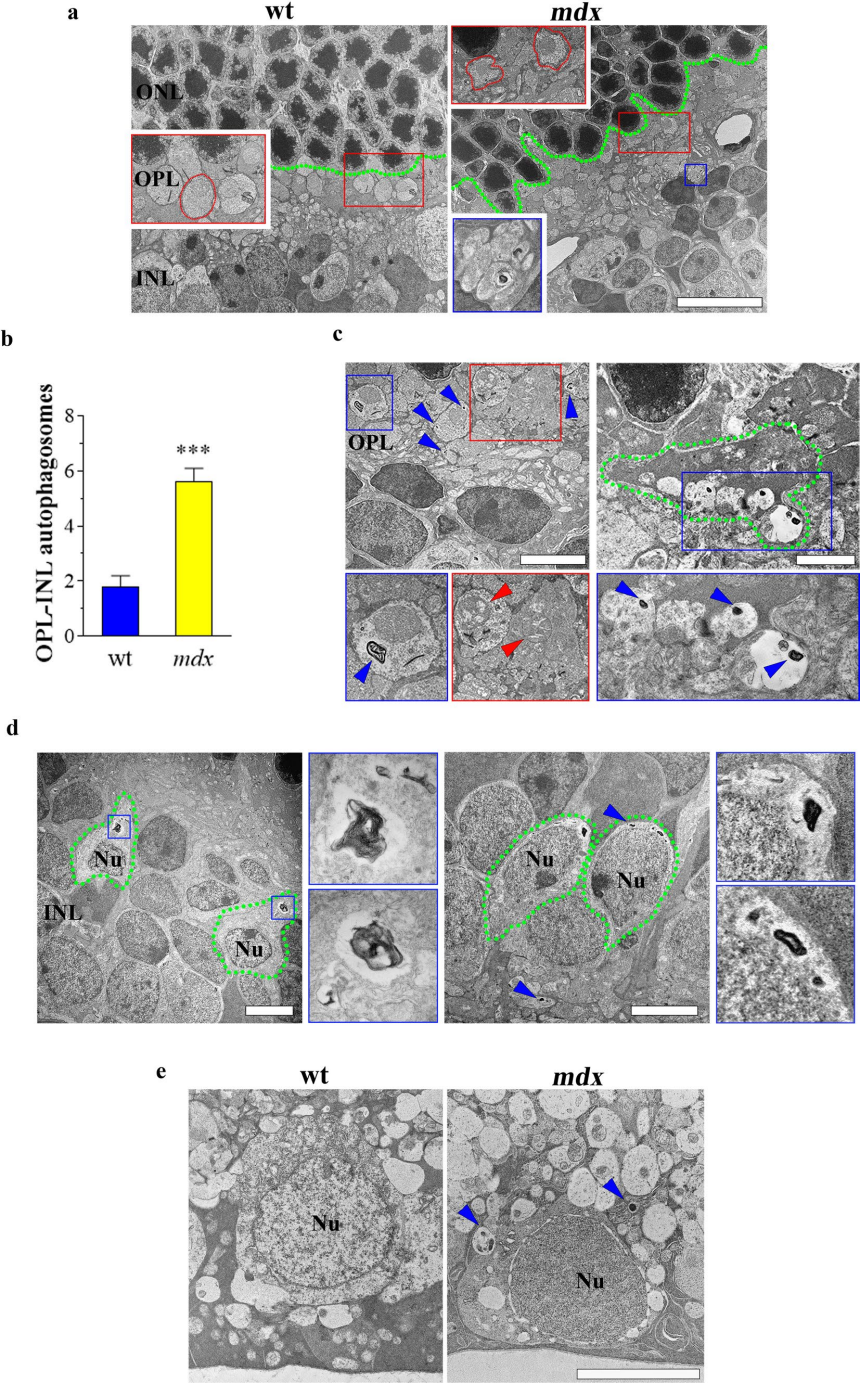
Ultrastructure of dystrophic mouse retinas by TEM. **a** Ultrathin sections of wt and *mdx* retinas. Green dotted line defines the ultrastructural organization of ONL, evidencing a disorganized arrangement in *mdx*. Insets represent enlarged image details: alteration in shape of photoreceptor synapses are focused in red inserts (red dotted lines); blue insert highlights the presence of autophagosomes in *mdx* retina. Scale bar: 10 µm. **b** The number of autophagosomes in OPL-INL was counted. Results are expressed as mean number of autophagosomes in each TEM image. ***p < 0.0001 vs wt (unpaired Student’s t test). **c** Ultrathin sections of *mdx* retina (OPL) showing alterations in rod spherules and cone pedicles. Blue arrowheads indicated the autophagosomes. Insets represent enlarged image details: blue insets focus the presence of numerous autophagosomes in photoreceptor synapses; red inset and red arrowheads indicate mitochondrial damages. Green dotted line highlights an altered single cone pedicle. Scale bars: 5 µm (left panel) and 2 µm (right panel). **d** Ultrathin sections of *mdx* retina (INL) showing the presence of autophagosomes in bipolar (left panel) and amacrine (right panel) cells, circled by green dotted lines. Autophagosomes are indicated by blue arrowheads or are highlighted in the blue insets representing enlarged image details. Scale bars: 5 µm. **e** Ultrathin sections at GCL level of wt and *mdx* retina, this latter displaying ganglion cells with autophagosomes at different stages of maturation (blue arrowheads). Scale bar: 5 µm. Nu: nucleus. Images and quantitative data are representative of *n* = 6 retinas from different mice

To examine whether the loss of Dp427 deeply affects OPL-INL and GCL, we performed TEM analyses on the retina of young adult *mdx* mice. We did not observe extensive cell death features at ultrastructural levels. However, as shown in Fig. 4a, ONL strata of *mdx* retinas looked impaired when compared to wt mice, as photoreceptors architecture appeared less packed. In addition, photoreceptor synapses were altered in shape and located in the unpacked area of ONL, thus conferring to OPL strata a disorganized arrangement. In *mdx* mice, many rod spherules appeared deeply modified in their global aspect as they lose their regular profile and, to note, numerous vacuoles with double or multiple-membrane were detected in the whole OPL thickness and in the cytoplasm of several cell body in the INL. The number of these autophagosomes containing electron-dense materials was significantly higher in *mdx* than wt retinas (Fig. 4b). Higher magnification analysis of *mdx* first synapse confirmed the existence of many autophagosomes both in rod spherules and in cone pedicles (Fig. 4c). In particular, vesicles in cone pedicles localized mostly on the invaginating synapses at their flat base, likely into dendrites of cone bipolar cells. Also, photoreceptor terminals exhibited damaged mitochondria showing loss of mitochondrial cristae. Based on the shape and the position, the autophagosomes in the INL of *mdx* retinas belong to bipolar and amacrine cells (Fig. 4d). Finally, different ganglion cells containing autophagosomes were detected in in GCL of *mdx* mice while their presence was scarce in wt retinas (Fig. 4e). Of interest, autophagosomes were found at different stages of maturation, containing electron-dense material or partially degraded contents.

### Eye features and autophagy in dystrophic models of *Drosophila melanogaster*

**Fig. 5:**
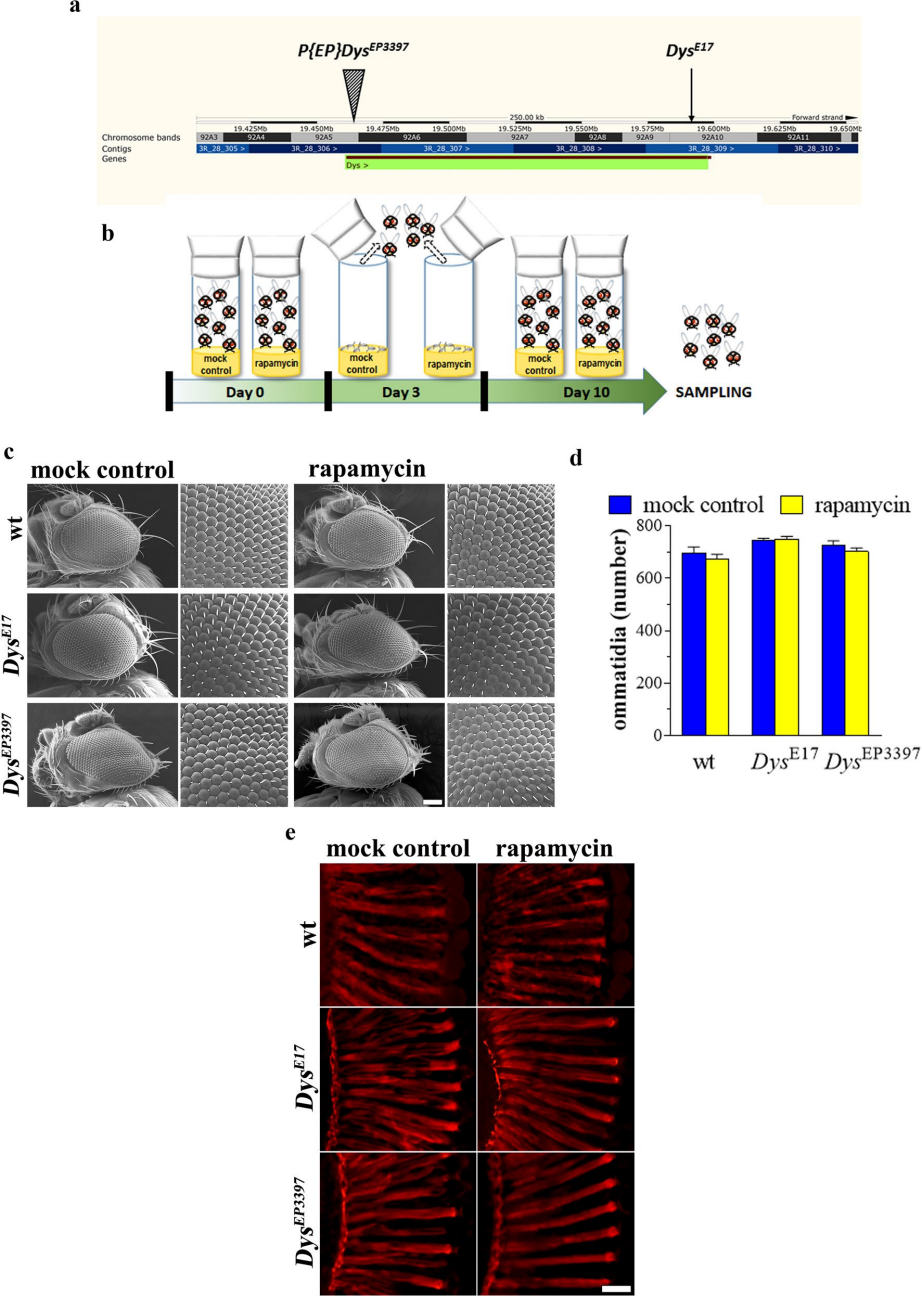
Dystrophic eye phenotype in *Drosophila melanogaster*. **a** Allele description of the *Dys*^E17^ and P{EP}*Dys*^EP3397^. The Drosophila dys gene (136204n) is shown as a green highlighted red bar extending on chromosome 3R:19,461,085..19,597,288 (BDGP6.28). The DysEP3397 allele is shown as a wedge representing an insertion of the transgenic transposon P{EP} on 3R:19,464,148, chromosome band 92A5. The DysE17 allele is a point mutation induced by ethyl methanesulfonate causing a nucleotide change C19590458T and consequently the amino acid change Q2807term|Dys-PA. It is shown as an arrow on 3R:19,590,458, chromosome band 92A10. **b** Experimental schedule on *D. melanogaster*. For mating and eggs laying flies (15 females and 10 males) were placed in vials containing standard diet supplemented with 10 nM rapamycin or with rapamycin vehicle as a control (mock treatment). At day 3, adults were removed and, at around day 10 from mating, the eclosed animals (young adults: 1–2 days of adult age) were sampled for analysis. **c** Images of Drosophila eye obtained by SEM for wt (Oregon-R) and dys mutants, i.e., *Dys*^E17^ and *Dys*^EP3397^ homozygous flies. Scale bar: 100 μm. Right panels depict magnificated images showing properly arranged ommatidia and bristles. **d** Quantitative analysis of ommatidia number. **e** Fluorescence microscopy analysis of fly eyes (longitudinal sections) stained with phalloidin to detect rhabdomere morphology. Scale bar: 20 μm. Images and quantitative data are representative of at least *n* = 30 animals obtained from 5 independent experiments

The visual systems of vertebrates and the fruit fly *D. melanogaster* share structural and functional characteristics [76]. During Drosophila development, eye discs contain ommatidia, composed of 8 photoreceptor neurons or retinula (R) cells. R1-R6 neurons may be thought of as rods and R7 and R8 neurons as cones [76]. The R1-R6 neurons project axons to and form synapses in the lamina, the first optic neuropile, while R7 and R8 axons, along with lamina neurons, arborize in distinct layers within the medulla. In terms of network and visual information processing, the vertebrate retina OPL is comparable to the fly lamina [76]. Drosophila dys is as complex as its mammalian counterparts since encodes three large-isoforms of dystrophin-like protein (DLP1, DLP2, and DLP3) and three truncated products (Dp186, Dp205 and Dp117) [77]. To test whether Drosophila DLP (orthologue of mouse and human dys) may have a role in the homeostasis of fly retina neurons, interacting with autophagy, we used different genetic loss-of-function homozygous mutants for dys, i.e., *Dys*^E17^and *Dys*^EP3397^ (Fig. 5a). The former is a null allele as a result of a nonsense mutation, that truncates the protein just before the WW domain mediating the interaction between dystrophin and dystroglycan [57]. *Dys*^EP3397^ is a null allele resulting from the insertion of the transposon P {EP}, 750 bp upstream of the DLP2 initiator ATG [59].

In the fly life cycle, fly larvae hatch from laid fertilized eggs and eat continuously, stopping only to molt twice after first instar and second instar stages. At 5/6 days after egg laying, third instar larvae leave the food and “wander” as they prepare to undergo metamorphosis into the adult fly (9/10 days). For these reasons *D. melanogaster* is a suitable organism for in vivo chronic drug delivery from fertilized eggs to adult stages. As shown in Fig. 5b, Oregon-R (wt strain), and homozygous *Dys*^E17^ and *Dys*^EP3397^ flies were raised in vials containing standard Drosophila control diet alone or mixed with 10 nM rapamycin, a widely used autophagy activator shown to be effective also in flies [78]. After eggs laying, mating flies were removed and the visual system of eclosed adults at around day 10 from mating (young adults: 1–2 days after eclosion) was analyzed. SEM analysis revealed that the external eye of wt and dys mutants has a similar phenotype with a highly regular arrangement of facets and normal mechanosensory bristles (Fig. 5c), along with a comparable number of ommatidia (Fig. 5d). The administration of rapamycin did not elicit any appreciable anatomic alteration on the external eye indicating the absence of toxicity and of eye-structure degeneration during larval development. Longitudinal section of fly eye revealed a regular length of rhabdomere stalks in *Dys*^E17^ and *Dys*^EP3397^ in comparison with wt (Fig. 5e), further excluding a severe structural damage in the absence of functional DLP. However, the internal eye of tested mutants exhibited clear signs of degeneration characterized by defective arrangement of rhabdomere columns and extensive vacuolization. Of interest, *Dys*^E17^ and *Dys*^EP3397^ phenotype recovered in the presence of rapamycin.

**Fig. 6:**
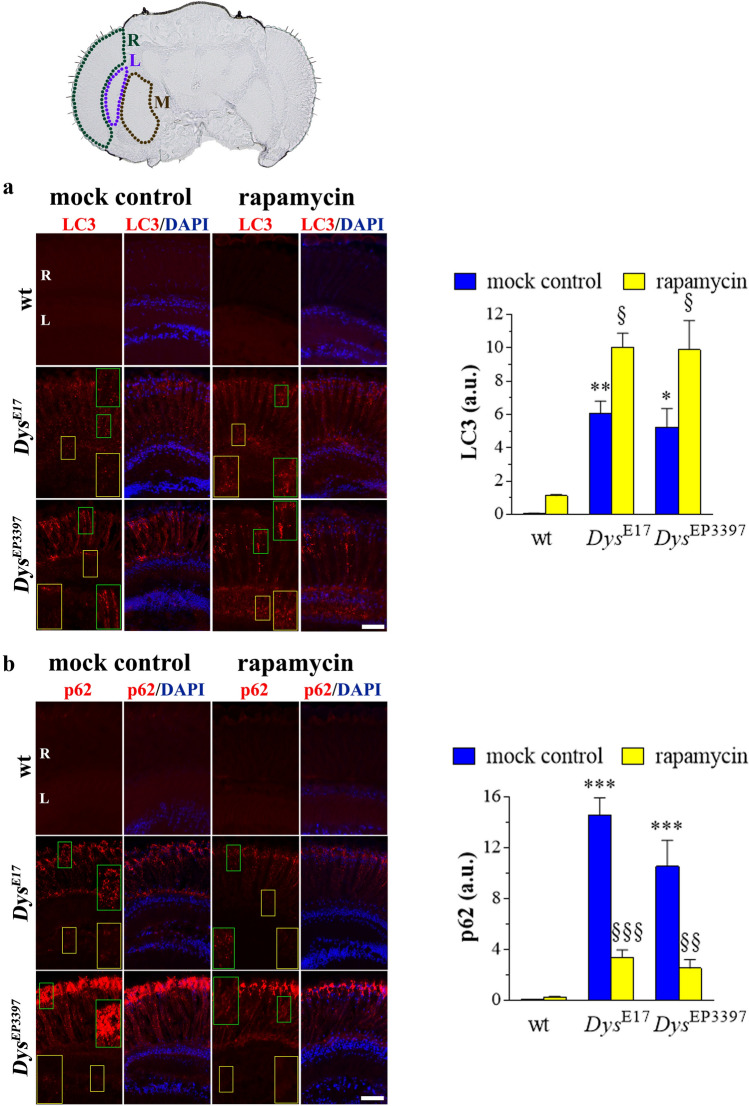
Retina autophagy in the dystrophic *Drosophila melanogaster*. The cartoon in the upper panel represents a section of Drosophila head at approximately the mid-brain. Limited areas depicted the major components of fly visual system: retina (R, green), lamina (L, purple), and medulla (M, brown). Both wt (Oregon-R) and dys mutants, i.e., *Dys*^E17^ and *Dys*^EP3397^ homozygous flies, were grown in standard diet supplemented with 10 nM rapamycin or with rapamycin vehicle as a control (mock treatment). Confocal immunofluorescence imaging of LC3 **(a)** and p62 **(b)** in the eye of young adults (1–2 days after eclosion) of Drosophila. DAPI was used for nuclei detection. Scale bar: 20 μm. Insets represent enlarged image details. Quantitative analysis of LC3 and p62 immunofluorescence was shown in the right graphs. Results are expressed as arbitrary units (a.u.). *p < 0.01, **p < 0.001, and ***p < 0.0001 vs wt; § *p* < 0.01, §§ *p* < 0.001, and §§§ *p* < 0.0001 vs the respective mock control (one-way ANOVA followed by the Tukey post-test). Images and quantitative data are representative of at least *n* = 30 animals obtained from 5 independent experiments

Confocal analysis of LC3 and p62 immunostaining revealed a large amount of LC3/p62 clusters in the retina and lamina of *Dys*^E17^ and *Dys*^EP3397^ homozygous flies (Fig. 6a,b), while their signal in wt flies was very weak. Accordingly, quantitative analysis showed a significant increase of LC3 and p62 staining in eyes sections of Drosophila mutants. Noteworthy, rapamycin increased further the expression/clustering of LC3 immunofluorescence in the retina and lamina of *Dys*^E17^ and *Dys*^EP3397^ mutants while p62 staining significantly decreased. These results confirmed that rapamycin treatment effectively reactivates autophagy in the mutant fly models, thus indicating a reinstatement of the autophagosomes turnover.

### Neuronal alterations and involvement of autophagy in the retina of dystrophic *Drosophila melanogaster*

**Fig. 7:**
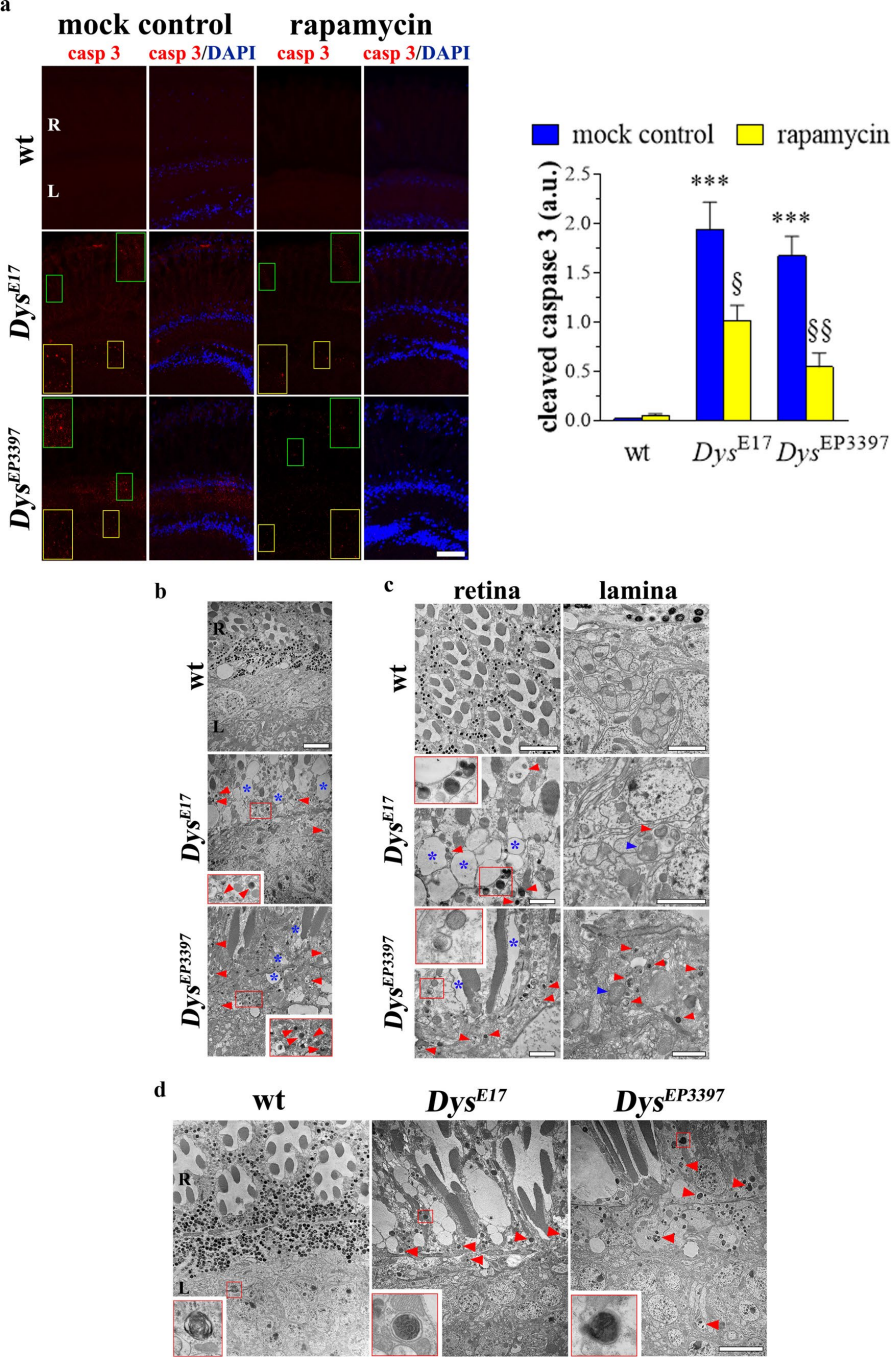
Retina neurodegeneration in the dystrophic *Drosophila melanogaster*. Both wt (Oregon-R) and dys mutants, i.e., *Dys*^E17^ and *Dys*^EP3397^ homozygous flies, were grown in standard diet supplemented with 10 nM rapamycin or with rapamycin vehicle as a control (mock treatment). **a** Confocal immunofluorescence imaging of cleaved (active) caspase 3 in the eye of young adults (1–2 days after eclosion) of Drosophila. DAPI was used for nuclei detection. Scale bar: 20 μm. Insets represent enlarged image details. Quantitative analysis of caspase 3 immunofluorescence was shown in the right graph. Results are expressed as arbitrary units (a.u.). ***p < 0.0001 vs wt; § *p* < 0.01 and §§ *p* < 0.001 vs the respective mock control (one-way ANOVA followed by the Tukey post-test). **b–c** Ultrastructure of young adults Drosophila eye by TEM, showing alterations in the morphology of retina (R) and lamina (L) in *Dys*^E17^ and *Dys*^EP3397^ mutants. In both compartments, numerous vacuoles (blue asterisks) and autophagosomes (red arrowheads) were observed. Damaged mitochondria with disorganized cristae (blue arrowheads) were also revealed. Red insets represent enlarged image details of autophagosomes. Scale bars: 5 µm (**b**), 5 µm (**c**, left panel of wt), 2 µm (**c**, right panel of wt), 2 µm (**c**, left and right panels of *Dys*^E17^), and 2 µm (**c**, left and right panels of *Dys*^EP3397^). **d** TEM observations of wt, *Dys*^E17^ and *Dys*^EP3397^ eyes of flies treated with rapamycin. Fly mutants recovered the normal morphology in comparison with wt and exhibit numerous autophagosomes (red arrowheads) in both retina (R) and lamina (L). Red insets represent enlarged image details of autophagosomes. Scale bar: 5 µm. Images and quantitative data are representative of at least *n* = 30 animals obtained from 5 independent experiments

We next investigated whether DLP alterations and autophagy impairment somewhat associated with retina neurodegeneration. As shown in Fig. 7a, very faint cleaved caspase 3 immunostaining was detected in the eyes of wt Drosophila young adults, while a significant increase of the sparse dot-like caspase 3 signals was observed in both *Dys*^E17^ and *Dys*^EP3397^ homozygous mutants. In particular, apoptotic neurons were found mostly in the lamina. Of interest, the administration of 10 nM rapamycin to Drosophila dys mutants clearly reduced the immunostaining of active caspase 3.

Accordingly, TEM ultrastructural analysis of degenerative phenotypes of *Dys*^E17^ and *Dys*^EP3397^ showed a marked alteration in the morphology of retina and lamina when compared to wt flies (Fig. 7b,c). Numerous vacuoles were observed in the retina inside and between rabdomeres producing a general loss of normal architecture. Spread vacuolization was also detected in the lamina. Furthermore, numerous accumulated autophagic vacuoles were evident both in the mutant retina and lamina as well as the presence of damaged mitochondria with disorganized cristae, typical of apoptotic neurons. As expected, even more autophagosomes were detected in both *Dys*^E17^ and *Dys*^EP3397^ flies treated with rapamycin (Fig. 7d); similar effects were achieved also in wt eyes. The enhancement of autophagy paralleled the evident morphological recovery of Drosophila mutants since the retina/lamina structure of rapamycin-treated *Dys*^E17^ and *Dys*^EP3397^ flies had features identical to the wt.

### Visual deficit and involvement of autophagy in dystrophic *Drosophila melanogaster*

**Fig. 8:**
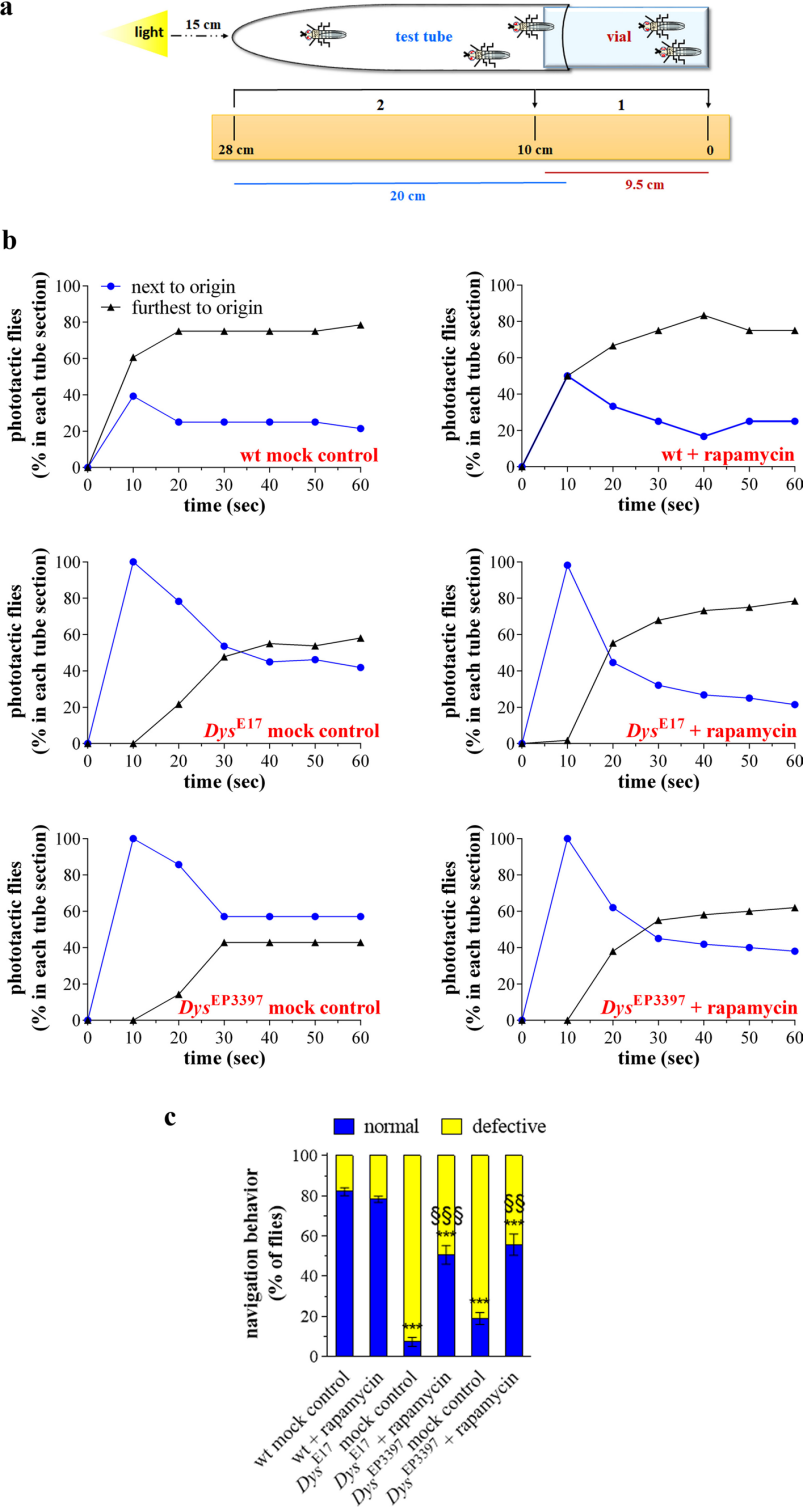
Visual function in the dystrophic *Drosophila melanogaster*. Both wt (Oregon-R) and dys mutants, i.e., *Dys*^E17^ and *Dys*^EP3397^ homozygous flies, were grown in standard diet supplemented with 10 nM rapamycin or with rapamycin vehicle as a control (mock treatment). **a** Experimental procedure to analyze the visual response of flies. Young adults (1–2 days after eclosion) of *D. melanogaster* (10–20 flies) were introduced in the dark in a transparent apparatus (2.5 × 28 cm; plastic vial inserted and connected to a glass tube) placed horizontal and perpendicular (15 cm away) to the light source. After 30 min the apparatus was gently pounded down to place the flies at opposite end from the light. The light was then turned and a camera was recording fly behavior and their movement towards the light source. The experiment ended after 1 min. **b** Analysis of Drosophila phototaxis. Young adults flies were counted every 10 s in each half of the apparatus, i.e., 0–10 cm (the chamber nearest to origin) and 10–28 cm (the chamber furthest to origin). Results are expressed as the percentage of total flies in the chambers at each time points. **c** Analysis of Drosophila navigation strategies during phototaxis assay. Results are expressed as percentage of flies exhibiting a normal or defective behavior within each experimental group. Defective behavior: ***p < 0.0001 vs wt; §§ *p* < 0.001 and §§§ *p* < 0.0001 vs the respective mock control (two-way ANOVA and Sidak’s multiple comparisons test). Data are representative of at least *n* = 30 animals obtained from 3 independent experiments

The climbing ability of wt, *Dys*^E17^, and *Dys*^EP3397^ flies immediately after eclosion was almost comparable. Indeed, in the negative geotaxis assay, the apparent different performance of *Dys*^E17^ and *Dys*^EP3397^ was not statistically different vs wt (Suppl. Fig. 2). Accordingly, using different stocks, it has been shown previously that Drosophila dys mutants begin adult life with normal mobility opposed to gravity [22]. To further demonstrate that the autophagy impairment observed in the absence of functional DLP altered the visual system of the *D. melanogaster*, phototaxis assay was performed with young adult flies (1–2 days after eclosion) of the different genotypes. Drosophila exhibits a positive phototactic behavior preferring light-exposed than shaded areas when given a choice. It is important to note that the visual observation of wt, *Dys*^E17^, and *Dys*^EP3397^ young adults in vials under standard conditions did not reveal obvious differences in the horizontal motile behavior, like it is needed during the phototaxis assay. In our experiments, young adults of *D. melanogaster* were introduced in the dark in a test apparatus placed away from a perpendicular light source (Fig. 8a). The light was then turned on and the flies were counted every 10 s in each half of the apparatus. As shown in Fig. 8b, after 20 s almost 80% of wt flies have moved towards the second chamber showing that they were attracted from the light. Similar behavior was found in the presence of rapamycin. In contrast, an important fraction of *Dys*^E17^ and *Dys*^EP3397^ population of flies did not reach the light within 1 min (ca. 58 and 43%, respectively) and they do it more slowly. These results indicated that the decreased responsiveness to the light of fly mutants is a consequence of vision defects, although we cannot exclude a role, at least in part, of a decreased mobility due to muscular defects. Consistent with the recovery of the eye homeostasis, the administration of rapamycin ameliorated robustly the light response of *Dys*^E17^ mutants reaching values comparable to wt, whereas the drug response of *Dys*^EP3397^, although positive, was less pronounced (Fig. 8b).

As further index of vision response we assessed the navigation behavior of Drosophila during phototaxis assay. In particular, flies that move straight towards the light source have a normal behavior while motionless animals, those moving perpendicular to the light source or unbiased towards and away from the light source have a defective behavior. The number of *Dys*^E17^ and *Dys*^EP3397^ animals with defective navigation was significantly higher when compared to wt (Fig. 8c). Of interest, the autophagy enhancement by rapamycin partially recovered the navigation behavior of mutant flies, increasing their responsiveness to the light.

## Discussion

Pre-clinical and clinical observations suggested that mutations affecting dys, including the long dys products, alter retinal homeostasis and predispose DMD patients to retinal complications [9, 14, 22, 32–46]. However, the mechanisms underlying the fact that full-length dystrophin Dp427 alterations trigger the deterioration of retinal function remain elusive. This study demonstrates that full-length dystrophin in neurons is required for proper autophagy turnover, thus maintaining retinal functions. Also, we provide proof of concept of the therapeutic potential of autophagy tuning for defective dystrophin-induced neurodegeneration.

Increasing evidence shows that autophagy is involved in retinal physiology and pathology and that defective autophagy contributes to retinal degeneration [48, 79, 80]. Of interest, upregulated autophagy in the neuro-retina affects the apoptosis/autophagy cross-talk favoring cell survival [51, 52]. We showed that, albeit *mdx* retinas do not present a severe phenotype, in agreement with previous observations [39, 44], physiological levels of Dp427 are likely needed for retinal homeostasis, since different neuronal populations are somewhat apoptotic. In addition, cell death and impaired autophagy overlaps in retinal neurons of *mdx* mice. In other words, neurons committed to die by apoptosis, localized mainly to the OPL-INL and to the GCL, have an altered autophagic turnover, i.e., a block of the autophagic flux.

Dp427 is present in photoreceptor, bipolar cells (mostly ON-type) and amacrine cells [34, 35, 38, 39, 44] and may therefore play a major role at the first synapse. Consistently, dystrophin signal colocalizes with ON-bipolar dendritic tips in the OPL of mice retinas where presynaptic DGC is shown to regulate the synapses between photoreceptor and bipolar cells [46]. In mice, Dp427 is proportionally more expressed in synapses between cones and cone bipolar cells compared with those formed between rods and rod bipolar cells, being the most abundant dys produced in INL [38, 44]. An important signal for Dp427 is detected also in the GCL [44]. In agreement with the expression pattern of Dp427, we found that the autophagy dysfunction in *mdx* retinas is present at photoreceptor axonal terminals (CtBP2 positive) that make synapses with dendritic tips (mGluR6 positive) of ON-type bipolar cells (MAb115A10 positive), including the rod bipolar cells (PKC positive). The fact that MAb115A10 positive cells in INL consistently express LC3, together with the expression of Dp427 in cone ON bipolar cells [38], strongly suggests that apoptosis/autophagy unbalance affects also the ON-type cone bipolar cells. In addition, the presence of autophagy defective and MAb115A10/calbindin negative cells in the INL of *mdx* retinas indicates that horizontal cells do not change in the absence of Dp427, also providing an indirect argument for the involvement of the cone OFF bipolar cells. In this respect, based on their localization in the mid-distal INL, the LC3 positive/MAb115A10 negative neurons are likely to belong to OFF-type bipolar cells. LC3 immunostained cells localize also to the proximal INL of *mdx* mice, and therefore are amacrine cells. Regarding the type of amacrine cells that these may represent, the classic localization pattern of cholinergic amacrine neurons (two mirror-symmetric populations in the proximal INL and in the GCL) is not consistent with the observed localization of LC3. In contrast, our data show that a number of GABAergic (GAT-1 positive) and glycinergic AII (Dab-1 positive) amacrine cells are affected in the absence of the full-length dystrophin Dp427. Finally, the reliable colocalization of LC3 with ß-tubulin III in the GCL, indicates the involvement of ganglion cells in autophagy impairment of *mdx* retinas. These results are confirmed at ultrastructural level showing altered retina architecture and damaged cells, i.e., bipolar, amacrine, and ganglion cells, with accumulated autophagosomes in OPL-INL and GCL. Noteworthy, the absence of Dp427 compromises, at least in part, both the pre-synaptic photoreceptor terminals (rod spherules and cone pedicles) and their post-synaptic sites (dendrites of bipolar cells) indicating the key role of full-length dystrophin in stabilizing the structure of the first synapse, a highly sophisticated and conserved chemical synapse in visual signal processing, specialized for the fast and continuous release of glutamate onto postsynaptic cells [75]. This structural defects caused by mutated Dp427 may underlie the abnormality of the ERG wave reported recently in young adult *mdx* mice, i.e., the minor changes in scotopic a- and b-wave amplitudes and normal photopic ERGs of *mdx* mice compared to littermate controls [44] and suggests the key role of full-length dystrophin to preserve retinal functions and the physiological autophagy turnover. Defects of full-length dystrophin slightly impair ERG responses in DMD patients [9]. In addition, ERG responses in the absence of Dp427 worsen in mice with ageing and ischemia, and dysfunctional Dp427 is associated with vaso-proliferative retinopathy [44].

Clinical studies suggest that emotional disturbances are common in DMD patients with mutations that specifically impede expression of Dp427 dystrophin [6]. Accordingly, the loss of full-length dystrophin in mice alters the functioning of the neuronal circuit of fear, and this may impact on behavioral tests [30, 81], as for instance visually guided performance [82]. In this study we thus analyzed the visual system of *D. melanogaster* as it is considered a very potent in vivo tool to study human neurodegenerative diseases, including those affecting retina [83–85]. Of interest, Drosophila proved most useful as a model organism of muscular dystrophy (and DMD) [11–13, 58] and dys fly mutants were used to study the role of dystrophins in the nervous system [14, 21–23]. In the developing eye of Drosophila 3^rd^ instar larvae, the expression of dys transcripts is reported in the neuropile, the optic lobes and the axons of photoreceptor neurons while in the adult dystrophin localizes mainly to the lamina [14, 22]. Functionally, dystrophin and DGC are involved in the organized lamina plexus and are required for photoreceptor cell elongation and axon migration [14, 22]. Here we demonstrate that defective expression of DLPs does not elicit any appreciable anatomic alteration of the external eye phenotype of young adults of Drosophila although clear signs of degeneration occurs in rhabdomeres. Structural damages are evident in the internal network of retina/lamina where photoreceptors make the first synapse. In particular, both dysfunctional autophagic flux, i.e., the accumulation of autophagosomes, and apoptotic features are detected in the eye neurons with defective DLP. Noteworthy, the reactivation of the autophagosome turnover by rapamycin prevents, at least in part, the neuronal cell death, also efficiently counteracting the neuron structural changes (and their synaptic connections) induced by mutant dys. Also, the visual system of the dystrophic *D. melanogaster* is impaired functionally since the young adults mutant flies exhibit decreased responsiveness to the light without significant mobility defects, at least within our experimental protocol. Interestingly, sustained autophagy ameliorates their vision response. These findings demonstrate that unbalanced autophagy turnover is responsible for retinal damage and functional alterations caused by defective full-length dystrophin. Previous reports in *D. melanogaster* suggest that cell death-suppressing and differentiating effects of autophagy are required for eye formation [86]. In addition, defects in the autophagic pathway of Drosophila photoreceptors are shown at the basis of retinal degeneration [87].

In summary, combining analyses of different dystrophic models, this study demonstrates for the first time that full-length dystrophin plays a pivotal role in the homeostasis of neuro-retina. It is required for synapsis stabilization and neuronal survival, allowing also proper autophagy as a prerequisite for physiological cell fate and visual properties of the retina. Noteworthy, our data highlight that the connectivity between photoreceptors and bipolar neurons is affected by defective dystrophin, as well as amacrine and ganglion cells. Moreover, we show that apoptotic cell death and visual defects can ameliorate by restoring the autophagy turnover. With increased life expectancy and concomitant cardiovascular defects in DMD patients, the incidence of secondary neurodegenerative disorders, as for instance DMD-associated retinopathies, is expected to increase as well. The chain of molecular events downstream to mutated dystrophin and their therapeutic potential is worthy of further exploration in the damaged DMD retina. Generally, the retina is known as an extension of the nervous system and this allows the translation of eye research to brain physiopathology. In this respect, the strong association at molecular and cellular level between altered autophagy/apoptosis and neurodegeneration in the presence of dysfunctional dystrophins may open new avenues for neuroprotective intervention strategies.

## Electronic supplementary material

Below is the link to the electronic supplementary material.

Supplementary file1 (PDF 206 kb)

## Abbreviations

BSA: Bovine serum albumin
CtBP2: Synaptic ribbon marker C-terminal-binding protein 2
Dab-1: Disabled-1
DAPI: 4′,6-diamidine-2′-phenylindole dihydrochloride
DGC: Dystrophin-glycoprotein complex
DLPs: Dystrophin-like proteins
DMD: Duchenne muscular dystrophy
Dp427: 427-KDa dys protein
dys: Dystrophin
ERG: Electroretinography
GAT-1: γ-Aminobutyric acid transporter-1
GCL: Ganglion cell layer
GFAP: Glial fibrillary acidic protein
INL: Inner nuclear layer
IPL: Inner plexiform layer
LC3: Light-chain 3
mGluR6: Metabotropic glutamate receptor 6
ONL: Outer nuclear layer
OPL: Outer plexiform layer
PB: Phosphate buffer
PKC: Protein kinase C
R cells: Retinula cells
SEM: Scanning electron microscopy
TEM: Transmission electron microscopy
wt: Wild-type
