# Supplementary material for: Defects of full-length dystrophin trigger retinal neuron damage and synapse alterations by disrupting functional autophagy
Source: Cell Mol Life Sci. 2020 Aug 4;78(4):1615–36. doi: 10.1007/s00018-020-03598-5 (PMC7904721; doi:10.1007/s00018-020-03598-5)
Supplement: Supplementary file 1 — Supplementary file1 (PDF 206 kb) [file 18_2020_3598_MOESM1_ESM.pdf]

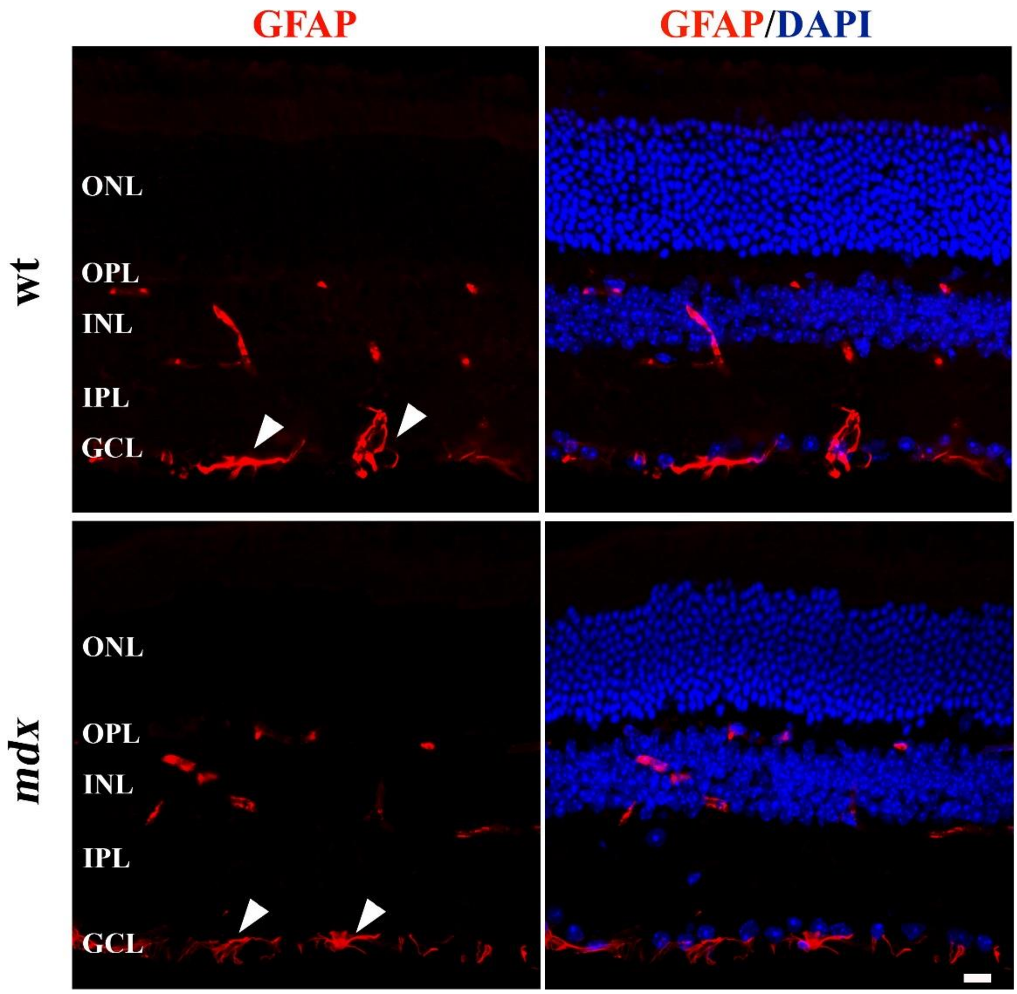

Supplementary Figure 1 Absence of gliosis in dystrophic mouse retinas. Confocal immunofluorescence imaging of GFAP in wt and *mdx* retinas. DAPI was used for nuclei detection. Astrocytes are indicated by white arrowheads. Non-specific staining to the vessels was detected in INL. Scale bar: 20 µm. Images are representative of n = 6 retinas from different mice

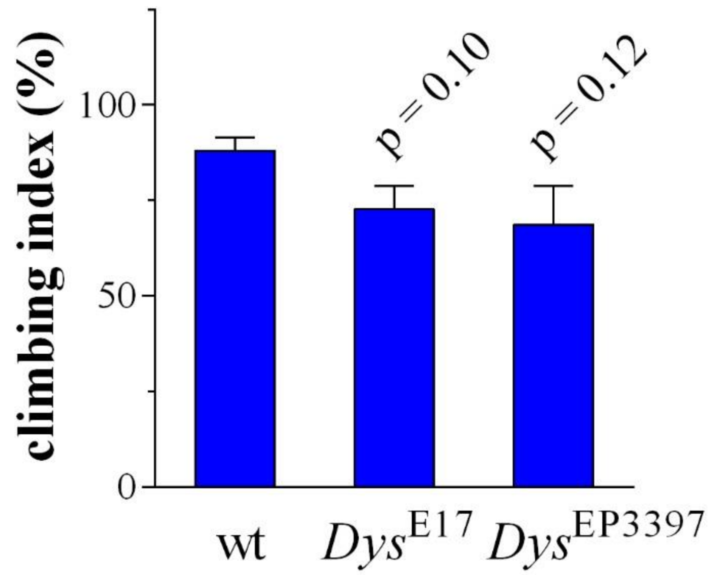

Supplementary Figure 2 Climbing index of dystrophic *Drosophila melanogaster*. Results are expressed as the percentage of young adult flies (1-2 days after eclosion), both wt (Oregon-R) and dys mutants (*Dys*^{E17} and *Dys*^{EP3397} homozygous flies), that climbed up to the 18 cm mark of the vial after 120 sec. The p-values *vs* the wt are reported (one-way ANOVA followed by the Tukey post-test). Data are representative of at least n = 50 animals obtained from 5 independent experiments.
